# Phase 3 program of evenamide, a glutamate release modulator, as add-on in patients with treatment-resistant schizophrenia: design of a potentially pivotal, international, randomized, double-blind, placebo-controlled trial

**DOI:** 10.1192/j.eurpsy.2025.829

**Published:** 2025-08-26

**Authors:** R. Anand, A. Turolla, G. Chinellato, R. Giuliani, F. Sansi, R. Hartman

**Affiliations:** 1 R&D, APC, St Moritz, Switzerland; 2 R&D, Newron Pharmaceuticals SpA, Milan, Italy; 3 R&D, NeurWrite LLC, Morristown, United States

## Abstract

**Introduction:**

Treatment-resistant schizophrenia (TRS) develops in ˜30% of patients, resulting in higher hospitalization rates, morbidity, mortality, and suicidality, and increased costs (Pompili et al CNS NDDT 2017; 16 870-884). Despite inconsistent findings of its efficacy, antipsychotic polypharmacy (APP) is frequently prescribed in an attempt to treat refractory symptoms (Correll ClinNorthAm 2012; 35 661-681). While marketed APs all act through the modulation of 5HT/DA transmission, clozapine, the only drug approved for TRS, seems to act on multiple receptor systems (Brunello et al NPP 1995; 13 177-213). The need to modulate non-monoaminergic targets is supported by findings of excessive glutamatergic activity, rather than increased dopamine synthesis, in patients with TRS (Demjaha et al BioPsy 2014; 75 11-3). Evenamide, devoid of biological activity at >150 CNS targets, is able to normalize excessive glutamate release without affecting basal levels. Evenamide has demonstrated benefits in several animal models of psychosis (monotherapy and add-on to AP). Benefit of evenamide as add-on was demonstrated in a phase 2, open-label trial (Anand et al IJNPP 2023; 174 216-229) and in a phase 2/3 randomized, double-blind study in patients not responding adequately to SGAs.

**Objectives:**

Evaluate the efficacy, safety, and tolerability of evenamide 30 mg bid as add-on treatment in patients with documented TRS receiving AP treatment but not adequately benefitting from a stable therapeutic dose.

**Methods:**

This is a prospective, potentially pivotal, phase 3, randomized, double-blind, placebo-controlled, 1-year international study, with a primary efficacy endpoint at 12 weeks and long-term efficacy endpoints at 26 and 52 weeks. Eligible patients must have a diagnosis of TRS according to the TRRIP consensus guidelines (Howes et al AmJPsy 2017; 174 216-229). During the 6-week screening period and throughout the study, adherence to background AP(s) and evenamide will be confirmed through measurements of plasma levels. Selection criteria include CGI-S of mildly to severely ill (3-6); BPRS total score ≥45, with a score ≥18 on core symptoms of psychosis, and PANSS total score ≥70. An Independent Eligibility Committee will determine patients’ eligibility. Patients improving ≥20% on the BPRS or ≥1 category on the CGI-S during the screening period will be excluded. Efficacy (PANSS, CGI-S/C, Q-LES-Q-SF, PSP scales) and safety (vital signs, ECG, lab tests, physical/neurological/eye exams, ESRS-A, CDSS, C-SSRS) will be evaluated at regular intervals.

**Results:**

Results from this study will determine whether addition of evenamide 30 mg bid to APs is associated with clinically important benefit in TRS patients.

**Conclusions:**

Positive results would support the role of glutamate modulators for the optimal treatment of TRS.

**Disclosure of Interest:**

R. Anand Consultant of: AbbVie, Acadia, BiolineRx, Domain, Enkam,Erydel, Forest, Janssen, Hoffman La Roche, Lundbeck, Noema, Ono, Pfizer, UCB, Shire, Sigma-Tau, Takeda, Teva, A. Turolla Employee of: Newron Pharmaceuticals SpA, G. Chinellato Employee of: Newron Pharmaceuticals SpA, R. Giuliani Employee of: Newron Pharmaceuticals SpA, F. Sansi Employee of: Newron Pharmaceuticals SpA, R. Hartman Employee of: Newron Pharmaceuticals SpA

